# Role of the JP45-Calsequestrin Complex on Calcium Entry in Slow Twitch Skeletal Muscles*[Fn FN2]

**DOI:** 10.1074/jbc.M115.709071

**Published:** 2016-05-04

**Authors:** Barbara Mosca, Jan Eckhardt, Leda Bergamelli, Susan Treves, Rossana Bongianino, Marco De Negri, Silvia G. Priori, Feliciano Protasi, Francesco Zorzato

**Affiliations:** From the § Departments of Anaesthesia and Biomedicine, Basel University Hospital, Hebelstrasse 20, 4031 Basel, Switzerland,; ** Center for Research on Ageing and Translational Medicine and DNICS (Department of Neuroscience, Imaging, and Clinical Sciences), University G. d'Annunzio, 66100 Chieti, Italy,; ¶ Molecular Cardiology Laboratories Fondazione Salvatore Maugeri, Via Maugeri 10/10°, 27100, Pavia Italy,; ‖ Department of Molecular Medicine, University of Pavia, Pavia Italy, and; ‡ Department of Life Science and Biotechnology, University of Ferrara, Via Borsari 46, 44100, Ferrara, Italy

**Keywords:** calcium-binding protein, excitation-contraction coupling (E-C coupling), gene knockout, muscle physiology, sarcoplasmic reticulum (SR)

## Abstract

We exploited a variety of mouse models to assess the roles of JP45-CASQ1 (CASQ, calsequestrin) and JP45-CASQ2 on calcium entry in slow twitch muscles. In flexor digitorum brevis (FDB) fibers isolated from JP45-CASQ1-CASQ2 triple KO mice, calcium transients induced by tetanic stimulation rely on calcium entry via La^3+^- and nifedipine-sensitive calcium channels. The comparison of excitation-coupled calcium entry (ECCE) between FDB fibers from WT, JP45KO, CASQ1KO, CASQ2KO, JP45-CASQ1 double KO, JP45-CASQ2 double KO, and JP45-CASQ1-CASQ2 triple KO shows that ECCE enhancement requires ablation of both CASQs and JP45. Calcium entry activated by ablation of both JP45-CASQ1 and JP45-CASQ2 complexes supports tetanic force development in slow twitch soleus muscles. In addition, we show that CASQs interact with JP45 at Ca^2+^ concentrations similar to those present in the lumen of the sarcoplasmic reticulum at rest, whereas Ca^2+^ concentrations similar to those present in the SR lumen after depolarization-induced calcium release cause the dissociation of JP45 from CASQs. Our results show that the complex JP45-CASQs is a negative regulator of ECCE and that tetanic force development in slow twitch muscles is supported by the dynamic interaction between JP45 and CASQs.

## Introduction

Activation of skeletal muscle contraction is initiated by the propagation of the action potential deep into the muscle fiber by means of the transverse tubular system (T system) (1–3). T-tubule depolarization causes massive release of Ca^2+^ from the sarcoplasmic reticulum (SR)^2^ throughout the entire length of the muscle fiber by a process called excitation-contraction coupling (ECC). The release of calcium from the SR initiates muscle contraction, and the subsequent SR calcium uptake by the sarco(endo)plasmic CaATPAse (SERCA) induces muscle relaxation. A macromolecular complex comprising the α1-subunit of the L-type Ca^2+^ channel (dihydropyridine receptor, Ca_v_1.1), the ryanodine receptor (RyR1), and calsequestrin (CASQ) (3, 4) in the contact region between the T system and the SR membrane, support excitation-contraction coupling. In resting conditions RyR1s are closed because of the inhibitory activity of Ca_v_1.1 (5). Ca_v_1.1 acts as a voltage sensor and generates orthograde signals to RyR1 causing release of Ca^2+^ from the SR into the myoplasm (5). RyR1s not only receive orthograde signals from Ca_v_1.1 but also generate retrograde signals that are important for the activation of Ca_v_1.1 channel activity (6, 7). Although the interaction between L-type Ca^2+^ channels (Ca_v_1.1) and RyR1s in the sarcoplasmic reticulum is a key step in the Ca^2+^ signaling process leading to muscle contraction (8), the fine regulation of these two Ca^2+^ channels is also mediated by a variety of accessory proteins (9). Ca_v_1.1 associates with other proteins, *e.g.* the α2/δ, β1a, and γ subunits (10), and the functional role of these accessory polypeptides is still under intensive investigation. In addition, Ca_v_1.1 is modulated by other protein components such Stac3, Rem, and JP45 (11–13). The C terminus of JP45 is localized in the SR lumen and interacts with CASQ, whereas its 130-amino acid residue-long cytoplasmic domain interacts with the α-interacting domain within the loop connecting repeat I and repeat II of Ca_v_1.1 (14). The JP45-CASQs complex may be involved in a signaling pathway connecting the SR calcium level to Ca_v_1.1 channel activity.

In a previous study we showed that ablation of JP45 in a CASQ1 KO background causes in flexor digitorum brevis (FDB) fibers a robust excitation-coupled calcium entry (ECCE) via Ca_v_1.1, which supports tetanic calcium transients (12). The ability to maintain the calcium transient is conveyed by the ablation of JP45, because in FDB fibers from CASQ1 null mice the calcium transients induced by tetanic stimulation fade during the delivery of the action potentials. The difference of calcium transients between JP45-CASQ1 double KO (DKO) and CASQ1 null results in striking changes of the mechanical properties of intact muscles isolated from the two different mouse models. EDL and FDB muscles from CASQ1 null mice are unable to maintain peak force development during repetitive action potentials (15–18), whereas ablation of JP45 in a CASQ1 background rescues the ability to develop force during repetitive tetanic contraction (12). The effect on peak force development upon tetanic stimulation in both JP45-CASQ1 double KO and CASQ1 null is fiber type-specific. Peak tetanic force fades only in fast EDL muscles and not in slow soleus muscles from CASQ1 KO (15) and from DKO1.^3^ Fast twitch fibers of fast muscles such as EDL express the skeletal isoform of calsequestrin (CASQ1) only, whereas slow twitch fibers of soleus and EDL also express the cardiac isoform (CASQ2) of calsequestrin along with CASQ1 (15, 19). The different expression pattern of CASQs involved in calcium buffering in slow fibers may compensate for the ablation of CASQ1 and for the ablation of both JP45 and CASQ1.

In this study we assessed the role, if any, of the complex JP45-CASQ2 on calcium entry by investigating the biochemical and functional properties of muscle fibers from JP45-CASQ2 double KO (DKO2) and JP45-CASQ1-CASQ2 triple KO (TKO).

## Experimental Procedures

### 

#### 

##### Mouse Models

JP45 KO, CASQ1 KO, and CASQ2 KO mice were obtained as described by Delbono *et al.* (20), Paolini *et al.* (21), and Denegri *et al.* (22), respectively. Double and triple KO mice were generated by crossing to each other established JP45 KO, CASQ2 KO, CASQ1 KO lines backcrossed in C57BL6J.

##### In Vitro Muscle Strength Assessment

EDL and soleus muscles from 4–6-week-old mice were dissected and mounted onto a muscle testing setup (Heidelberg Scientific Instruments). Muscles were triggered by field stimulation with 15-V pulses of 0.5-ms duration. Tetanic contractures were obtained by a train of pulses delivered at 100 Hz, and force output was digitized at 4 kHz by using an AD Instruments converter. EDL and soleus were stimulated with a train of tetanic contractures (400- or 1100-ms duration) delivered at 0.27 Hz. To measure peak index, solei were stimulated with three trains of 1-min duration each of tetanic stimulation (100 Hz, 1100-ms duration) delivered at 0.27 Hz. After the first train, solei were incubated for 10 min in Tyrode's solution in which 1.8 mm CaCl_2_ was replaced with 100 μm La^3+^. Solei were then stimulated with a second train of tetanic contractures, and at the end of stimulation they were incubated for 10 min in Tyrode's solution containing 1.8 mm CaCl_2_. Absolute force was normalized to the muscle cross-sectional area = wet weight (mg)/length (mm) × 1.06 (mg/mm^3^) (23).

##### Biochemical Analysis of Total Muscle Homogenate and Total Sarcoplasmic Reticulum Membrane Fractions

Total muscle homogenate was prepared from solei. Briefly, 2.5 mg of wet muscle was homogenized in 1 ml of cracking buffer containing 10% glycerol, 5% β-mercaptoethanol, 2.3% SDS, 6 m urea, 62.5 mm Tris, pH 6.8, and separated in a urea/SDS-PAGE (24). Total SR membrane fraction was isolated from back and hind limb muscles. 20% (w/v) muscle homogenate was prepared by using a buffer containing 0.3 m sucrose and 5 mm imidazole plus a mixture of anti-proteases (Complete EDTA-free Protease Inhibitor Mixture Tablets, Roche Applied Science, catalogue no. 05056489001). The homogenate was sedimented at 3,000 × *g*_max_ for 10 min, and the resulting supernatant was centrifuged at 15, 000 × *g*_max_ for 20 min to remove the myofibrillar protein components. The 15,000 × *g*_max_ supernatant was then centrifuged for 60 min at 100,000 × *g*_max_ to isolate the total SR (microsomal) pellet (14). SDS-polyacrylamide electrophoresis and Western blot of total SR proteins were carried out as described by Anderson *et al.* (14). Western blots were stained with the following primary Abs: anti-RyR1 (Thermo Scientific, catalogue number MA3–925, 1:5,000), anti-SERCA1 (Santa Cruz, catalogue number sc-8093; 1:1,000), anti-SERCA2 (Santa Cruz, catalogue number sc-8095, 1:6,000), anti-Cav1.1 (Santa Cruz, catalogue number sc-8160, 1:1,000), anti-β1a (Santa Cruz, catalogue number sc-32079, 1:250), anti-JP45 (14), anti-CSQ1 (Sigma, catalogue number C0743, 1:3,000), anti-CSQ2 (Abcam, catalogue number Ab3516, 1:800), anti-calreticulin (Santa Cruz, catalogue number sc-6467, 1:200, Thermo Scientific -PA3-900, 1:1,000), anti-sarcalumenin (Thermo Scientific, catalogue number MA3-932, 1:1,000), anti-albumin (Bethyl Laboratories, catalogue number A90-134P; 1:80,000), followed by peroxidase-conjugated Protein G (Sigma P8170; 1:125,000) or peroxidase-conjugated anti mouse IgG (Fab Specific) Ab (Sigma A2304; 1:200,000). The immunopositive bands were visualized by chemiluminescence using the Super Signal West Dura kit from Thermo Scientific (Thermo Scientific 34076). Densitometry of the immunopositive bands was carried out by using Bio-Rad GelDoc 2000 (supplemental Fig. 1).

##### Isolation of FDB Fibers

All animals of 4 weeks old were killed by cervical dislocation according to procedures approved by the local animal care committee. FDB muscles were extracted and digested with 0.2% collagenase (*Clostridium hystoliticum* Type I, Sigma) in normal mammalian Ringer buffer (137 mm NaCl, 5.4 mm KCl, 0.5 mm MgCl_2_, 1.8 mm CaCl_2_, 0.1% glucose, 11.8 mm HEPES, pH 7.4 NaOH) for 1 h at 37 °C 5% CO_2_. Muscles were washed with DMEM (Dulbecco's MEM: Ham's Nutrient Mixture F-12), 10% fetal bovine serum (FBS) to inactivate collagenase activity. Fibers were then gently separated from tendons in pure DMEM starting from a large until a narrowest pipette tip. All fibers were placed on 13-mm-diameter glass coverslips previously coated with 1.5 μl of natural mouse laminin (1 mg/ml Invitrogen) and placed in the incubator for 1 h to settle down. Afterward the medium was replaced with DMEM supplemented with 1% penicillin/streptomycin, 10% FBS and placed overnight in a cell incubator.

##### Calcium Transients and Manganese Influx Measurements

FDB fibers were incubated for 20 min at room temperature (20–22 °C) in Ringer solution containing 10 μm low affinity calcium indicator (25) Mag-Fluo-4 AM (Invitrogen), 50 μm *N*-benzyl-*p*-toluene sulfonamide (Tocris), and 5% of the cell permeant Pluronic (Invitrogen). Fibers were then washed twice with fresh Tyrode's solution, and measurements were carried out in Tyrode's solution containing 50 μm *N*-benzyl-*p*-toluene sulfonamide. Measurements were carried out with a Nikon ECLIPSE TE2000-U inverted fluorescent microscope equipped with a 20× PH1 DL magnification objective. The light signals from a spot of 1-mm diameter of the magnified image of FDB fibers were converted into electrical signals by a photomultiplier connected to a Nikon Photometer P101 amplifier. Fluorescence signal was acquired by custom-made (RCS AUTOLAB) software and analyzed by PowerLab Chart5 and Origin.6 programs. Changes in fluorescence were calculated as Δ*F*/*F* = (*F*_max_ − *F*_rest_)/(*F*_rest_). Kinetic parameters were analyzed using Chart5 software. For Mn^2+^ influx measurements fibers were first incubated in buffer (137 mm NaCl, 5.4 mm KCl, 1.8 mm MgCl_2_, 0 mm CaCl_2_, 5 mm EGTA, 0.1% glucose, 11.8 mm HEPES, pH 7.4) containing 50 μm ryanodine (Tocris, Bioscience), and 50 μm *N*-benzyl-*p*-toluene sulfonamide at 37 °C, 5% CO_2_ for 50 min. Fibers were then incubated at 20 °C for 25 min in with Tyrode's buffer containing 1.8 mm CaCl_2_, 5 μm fura-2 AM, 50 μm *N*-benzyl-*p*-toluene sulfonamide, 50 μm ryanodine. A glass coverslip carrying adhered FDB fibers was placed onto a chamber that was mounted on an inverted fluorescent microscope Nikon Eclipse TE2000-U equipped with P101 photomultiplier. Fibers were excited at 360 nm and then stimulated with a 300-ms duration train of 40-V pulse (0.5-ms duration) delivered at 100 Hz. Recordings were made by perfusing fibers with Tyrode's buffer in which 1.8 mm CaCl_2_ was replaced with Mn^2+^ containing 50 μm ryanodine, 10 μm EGTA in the presence or in the absence of 50 μm nifedipine.

##### Pulldown Assay

Microsomal vesicles derived from rabbit skeletal muscle were prepared as previously described (26). Proteins were solubilized at a final concentration of 1 mg/ml in a buffer composed of 1% CHAPS, 200 mm NaCl, 50 mm Tris-HCl, pH 8.5, 1 mm dithiothreitol, and anti-proteases for 4 h at 4 °C under vertical rotation. Soluble proteins were obtained by centrifugation at 20,000 × *g* for 15 min at 4 °C and then were diluted 10 times with a buffer containing anti-proteases mixture, 50 mm Tris-HCl, pH 8.5, 1 mm DTT, and 1 mm Ca^2+^ or 1 mm EGTA and finally incubated for 2 h at 4 °C under vertical rotation with GST-JP45 fusion protein (C-ter) immobilized on glutathione-Sepharose beads. After low speed centrifugation, the beads were washed 3 times with buffer containing 50 mm Tris-HCl, pH 8.5, 1 mm DTT, and 1 mm Ca^2+^ or 1 mm EGTA. The proteins were separated by electrophoresis in a 10% polyacrylamide gel and then transferred onto nitrocellulose membranes. The proteins pulled down with JP45 were detected by immunoblot analysis using antibodies specific for CASQ1 and CASQ2 proteins.

##### Statistics

Statistical analysis was performed using the Mann-Whitney test for two independent populations or by Dunnett's multiple comparison ANOVA post test for multiple population by using GraphPad software package.

## Results

### 

#### 

##### Decrease of Isometric Force Development in JP45-CASQ1 Double KO Mice Occurs in Fast EDL Muscle but Not in Slow Soleus Muscles

**FIGURE 1.**
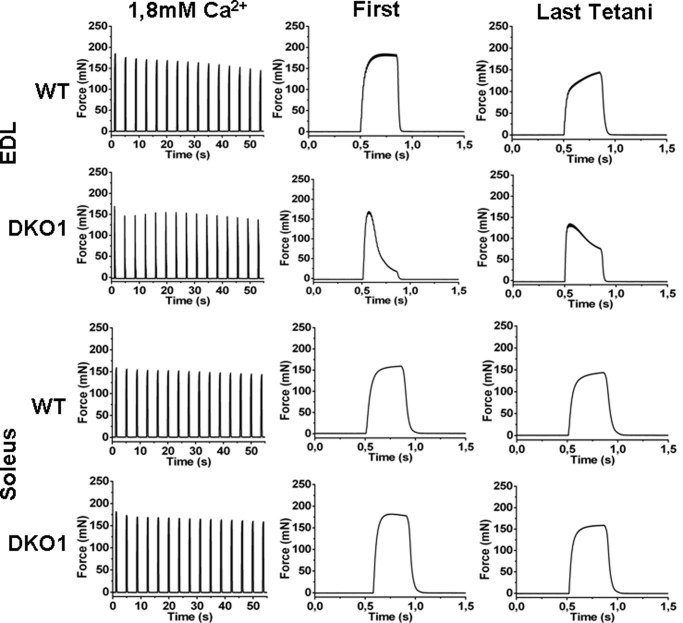
**Ca^2+^ transients and force generation in EDL and soleus muscles from WT and DKO mice.** *Left panels*, evaluation of tetanic force of intact EDL (*top two*) and soleus (*bottom two*) triggered by field stimulation in a bathing solution containing 1.8 mm CaCl_2_ with a train of tetanic stimulation (pulses of 0.5-ms duration at 100 Hz for 400 ms) delivered at 0.27 Hz (*left panels*). Time courses of force development of the first and last tetani of the trace are displayed in the *middle* and *right panels. WT*, wild type; *DKO1*, JP45-CASQ1 double knock-out. Representative traces of 6–9 muscles from 6 mice.

We compared the mechanical properties of intact fast (EDL) and slow (soleus) muscles from WT and JP45-CASQ1 double KO upon delivery of a tetanic stimulation of 400-ms duration (Fig. 1). The time course of tetanic contractures of EDL from DKO1 was dramatically different compared with those from WT mice. In EDL from DKO1 mice the train of repetitive pulses caused an initial increase of isometric force that then faded rapidly at the end of the tetanic stimulation (Fig. 1, EDL, *middle panel*, *First*) to ∼20% of the onset value. The reduction of force development of EDL is indicative of poor calcium store capacity of SR caused by the ablation of CASQ1, as a similar effect on force development was observed in EDL muscles from CASQ1 KO mice but not in the EDL muscles from JP45 KO mice, which have normal CASQ1 expression levels. The decay of force development in EDL muscles from JP45-CASQ1 double KO mice was partially reversed by stimulation with a train of tetani delivered at 0.27 Hz, a maneuver that activates a strong calcium influx which likely leads to replenishment of the intracellular calcium store (Fig. 1, EDL, *middle panel*, *Last Tetani*) (12). Tetanic contractions of 400-ms duration on soleus muscles from JP45-CASQ1 double KO show no difference compared with those of soleus from WT mice (Fig. 1, *Soleus*). This result is surprising as soleus muscles contains substantial fraction (55–60%) of type 2A/X fibers (Mosca *et al.* (Ref. 12)), *i.e.* fibers that should express CASQ1 only, whereby they should have a remarkable depletion of their calcium stores. The ability of soleus muscles from JP45-CASQ1 double KO mice to develop and maintain an isometric force during tetanic stimulation could be due to the expression of the CASQ2 isoform in slow twitch muscle fibers in addition to massive calcium entry triggered by the ablation of the JP45-CASQ1 complex. Thus, in the next set of experiments we tested the potential compensatory role of CASQ2 by comparing the skeletal muscle phenotype of JP45-CASQ2 DKO2 and JP45-CASQ1-CASQ2 TKO.

##### Protein Composition of Skeletal Muscle Sarcoplasmic Reticulum from WT, DKO2, and TKO Mice

**FIGURE 2.**
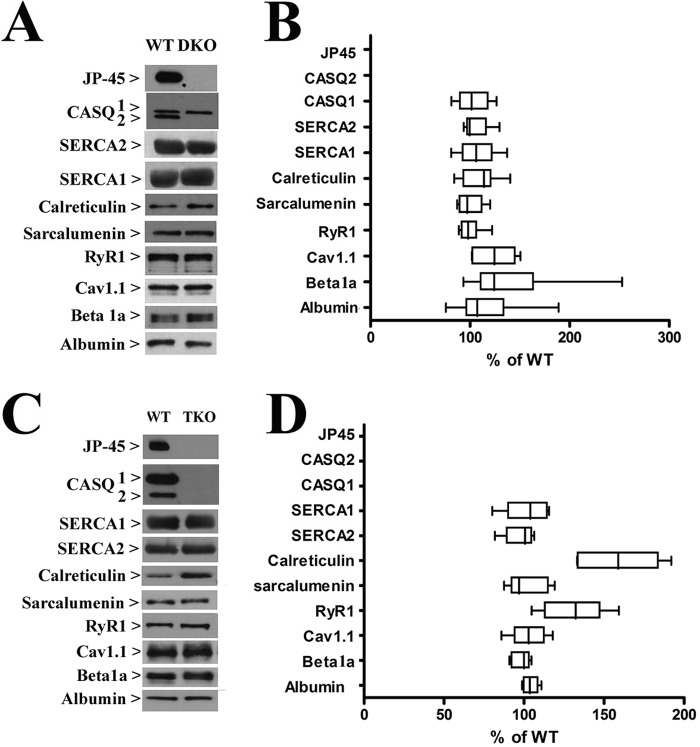
**Biochemical characterization of SR proteins from DKO and TKO mouse muscles.** *A*, representative immunoblots on SR preparations from DKO mice with the specified antibodies. *B*, we made two different SR preparations by pooling muscles from 4/5 mice for each preparation. By using both SR preparations, we then performed a total of six to nine determinations. Values were normalized with respect to intensity values obtained from control littermates, which were considered 100%. *C*, representative immunoblots on SR preparations from TKO mice with the specified antibodies. *D*, we made two different SR preparations by pooling muscles from four/five mice for each preparation. By using both SR preparations, we then performed a total of six to nine determination.

**FIGURE 3.**
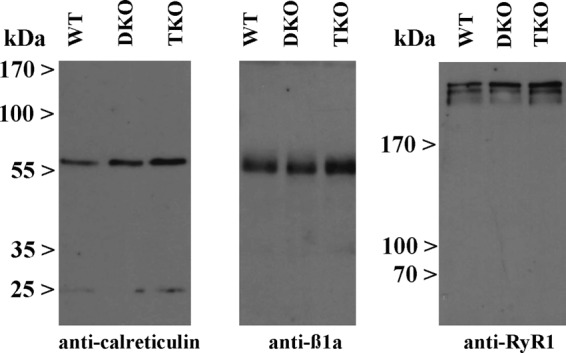
**Western blot of Soleus homogenates from WT, DKO2, and TKO mice.** Western blots of total homogenates of soleus muscles were probed with Abs against the proteins whose content changed in the total SR microsomal fraction. The panels show a representative blot that was performed three to five times by using two different total soleus homogenates.

The content of the main protein components of total SR membranes isolated from back and hind limb muscles of WT, DKO2, and TKO were examined by quantitative Western blot analysis (Fig. 2). We found no significant changes in the content of SERCA1, SERCA2, sarcalumenin, and albumin, a marker of T tubules membrane, in the total SR membrane fraction from WT and DKO2 and TKO. Ablation of CASQ1 in the JP45 null/CASQ2 null background caused a 60% increase (*n* = 8, *p* < 0.05, Mann-Whitney test) of calreticulin (Fig. 2, *panel D*). Total SR membrane fractions from TKO mice exhibited an ∼25% increase of RyR1 (*n* = 8, *p* < 0.05, Mann-Whitney test) (Fig. 2, *panel D*), whereas DKO2 showed no variation (Fig. 2 *panel B*). Ca_v_1.1 and Ca_v_β_1a_ expression levels did not change in TKO, whereas DKO2 exhibited a 25 and 56% increase, respectively (*p* < 0.05, paired *t* test) (Fig. 2 *panel B*). The soleus has a mixed fiber type composition, namely 55% are type 1 slow fibers, the remaining 45% are type 2A and 2X fast oxidative fibers (12, 15, 27, 29). This was the main reason why we decided to isolated total SR membranes from mixed back and hind limb muscles. Nevertheless, we performed Western blots with total homogenates isolated from solei and found comparable results. We probed Western blots of total homogenates of soleus muscles with Abs against the proteins whose content changes in the total SR microsomal fraction. Fig. 3 shows a representative blot that was performed 3–5 times by using two different total soleus homogenates. The data obtained with soleus total homogenate are consistent with those obtained with total SR microsomal fraction.

##### Calcium Transients in Single FDB Fibers from DKO2 and TKO Mice

**FIGURE 4.**
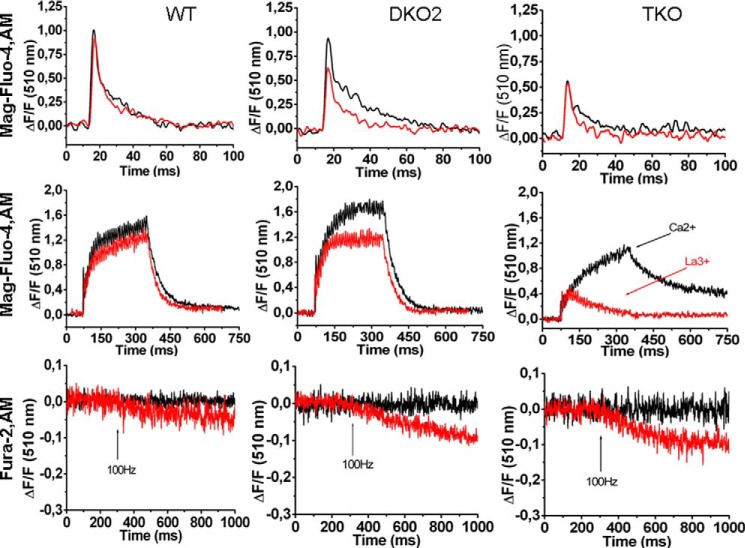
**Mag-Fluo-4 transients and quenching of fura-2 fluorescence.** *Top* and *middle panels*, single pulse (*top panel*) and tetanic stimulation (*middle panel*) of enzymatic dissociated FDB fibers from WT, JP45-CASQ2 DKO (DKO2), and JP45-CASQ2/CASQ1 TKO mice loaded Mag-Fluo-4AM. Single calcium transients were triggered by field stimulation with 40-V pulses of 0.5-ms duration. Tetanic calcium transient were triggers by a train of pulses delivered at 100 Hz for 300 ms. *Black traces* and *red traces* represent recordings carried out with Tyrode's containing 1.8 mm CaCl_2_ or 100 μm La^3+^, respectively. Representative traces of experiments on fibers were isolated from four to six mice of each genotype. *Lower panel*, fura-2 manganese quenching was carried out as described under “Experimental Procedures.” Single fibers were perfused with a Tyrode's solution in which 1.8 mm CaCl_2_ was replaced with manganese and then stimulated by a 300-ms duration train of pulses delivered at 100 Hz. *Red* and *black traces* represent recordings carried out in the absence and presence of 50 μm nifedipine in the extracellular buffer, respectively. Representative traces of experiments on fibers isolated from six mice of each genotype are shown.

We investigated resting Ca^2+^, and calcium transients evoked either by a single or by a train of action potentials in FDB fibers from WT, DKO2, and TKO (Fig. 4). The resting calcium fluorescence level measured with the ratiometric Ca^2+^ indicator indo-1 was 0.81 ± 0.09 (*n* = 75), 0.77 ± 0.07 (*n* = 40), and 0.81 ± 0.08 (*n* = 33) in WT, DKO2, and TKO mice, respectively (*F*_405_/*F*_480_ indo-1 ratio values are the mean ± S.D.). In the presence of 1.8 mm Ca^2+^ in the extracellular solution, the averaged peak intracellular Ca^2+^ transient induced by a single action potential in fibers from DKO2 was ∼8% lower compared with that of WT (0.91 ± 0.26, *n* = 58 *versus* 1.018 ± 0.18, *n* = 52, respectively; Δ*F*/*F_o_* values are expressed as mean ± S.D.) (Fig. 4, *upper traces*). Ablation of the CASQ1 calcium storage component in the JP45-CASQ2 null background caused a larger decrease of peak calcium (0.57 ± 0.18, *n* = 92; Δ*F*/*F_o_* values are expressed as the mean ± S.D.) (Fig. 4, *upper traces*). The half-time of the decay of the Ca^2+^ transients in FDB fibers from DKO2 (4.2 ± 1.6* ms, *n* = 100) and TKO mice (3.9 ± 1.7 ms, *n* = 92) was slower compared with WT (3.6 ± 0.70 ms, *n* = 52) (values are the mean ± S.D. *, multiple comparison Dunnett's ANOVA post test *p* < 0.05). Because there is no reason to believe that the pattern of expression of SERCAs in FDB muscles, which are made up of 80–90% type 2A fast oxidative glycolytic fibers and 10–20% of type I fibers (27–29), is different from that of back and hind limb skeletal muscles (Fig. 2, *panels B* and *D*), we assumed that the slower decay of the calcium transients is not consistent with a decreased calcium uptake by the SR because the SERCAs membrane content in DKO2 and TKO muscles fibers is not different from that of WT. The half-time for decay of calcium transient measured in the presence of 100 μm La^3+^ in the extracellular Tyrode's buffer showed no differences between WT (0.32 ± 0.7 ms, mean ± S.D., *n* = 37), DKO2 (3.5 ± 1.1 ms, mean ± S.D., *n* = 42), and TKO (2.8 ± 0.8 ms, mean ± S.D., *n* = 24). The summation of Ca^2+^ transient peaks evoked by a train of pulses delivered at 100 Hz in the presence of 1.8 mm Ca^2+^ in the extracellular solution in WT (1.52 ± 0.35, *n* = 43) was different compared with that measured in fibers from DKO2 (1.70 ± 0.43*, *n* = 92), and from TKO mice (1.09 ± 0.55*, *n* = 23) (Δ*F*/*F_o_* values are expressed as mean ± S.D. *, multiple comparison Dunnett's ANOVA post test *p* < 0.05) (Fig. 4, *middle traces*). In FDB fibers from JP45-CASQ2 double KO mice, La^3+^ caused a 20% decrease of the peak tetanic calcium transient when compared with that obtained in the presence of 1.8 mm CaCl_2_ (1.31 ± 0.41*, *n* = 36 *versus* 1.70 ± 0.43, respectively. Δ*F*/*F_o_* values are expressed as the mean ± S.D. *, unpaired *t* test *p* < 0.001) with no apparent decline of the plateau signal during tetanic stimulation (Fig. 4, *middle traces*, *middle panel*). It appears that the calcium influx component induced by the ablation of JP45 and CASQ2 is smaller compared with that obtained by the ablation of JP45 and CASQ1 (12). We are aware that slow fibers expressing both CASQ1 and CASQ2 represent 10–20% of the fiber types in FDB muscles (27). Thus, the 20% decrease of peak tetanic calcium transient in the presence of La^3+^ likely reflects the functional behavior of the fraction of type I fibers co-expressing both CASQ1 and CASQ2, which is present in our preparation. The block of calcium entry by La^3+^ during the tetanic calcium transient in fibers from TKO mice leads to a drastic change in the morphology of the tetanic calcium transients. After an initial peak having an amplitude 60% lower compared with that in the presence of 1.8 mm Ca^2+^ (0.46 ± 0.19* *versus* 1.09 ± 0.55, *n* = 23, respectively; Δ*F*/*F_o_* values are expressed as the mean ± S.D. *, unpaired *t* test *p* < 0.001) the calcium signal rapidly faded to base line toward the end of the tetanic stimulation (Fig. 4, *middle traces*, *right panel*). Such a response of FDB fibers from TKO mice in the presence of La^3+^ is similar but more pronounced compared with that of JP45-CASQ1 double KO mice (12).

##### Excitation-coupled Calcium Entry in FDB Fibers from WT, DKO2, and TKO Mice

On the basis of previous experiments, we believe that the calcium transients evoked by tetanic stimulation in FDB fibers from DKO2 and TKO mice are supported, at least in part, by the activation of excitation-coupled Ca^2+^ entry. This conclusion is supported by fura-2 manganese quenching experiments performed in FDB fibers (Fig. 4, *lower panels*). At an excitation wavelength of 360 nm, the Ca^2+^ independent isosbestic wavelength of fura-2, Mn^2+^ entry quenches fura-2 fluorescence (30). We measured the extent of the fluorescence quenching at the end of a 300-ms duration train of pulses delivered at 100 Hz. We found that Mn^2+^ quenching of fura-2 fluorescence was −0.032 ± 0.026 (*n* = 29), −0.059 ± 0.055*(*n* = 48), −0.081 ± 0.060^&^ (*n* = 29) for WT, DKO2, and TKO FDB fibers, respectively (Δ*F*/*F_o_* values are the mean ± S.D., multiple comparison Dunnett's ANOVA post test. *, *p* < 0.05; &, *p* < 0.01). The fura-2 fluorescence quenching by Mn^2+^ was abolished by the addition of 50 μm nifedipine (Fig. 4 *lower panels*, *black line*). Altogether, these data demonstrate that the increase of excitation-coupled Mn^2+^ entry in both DKO2 and TKO fibers is mediated by a nifedipine-sensitive voltage dependent calcium channel.

##### Enhancement of Excitation-coupled Calcium Entry Requires the Ablation of the JP45-CASQ Complex

**FIGURE 5.**
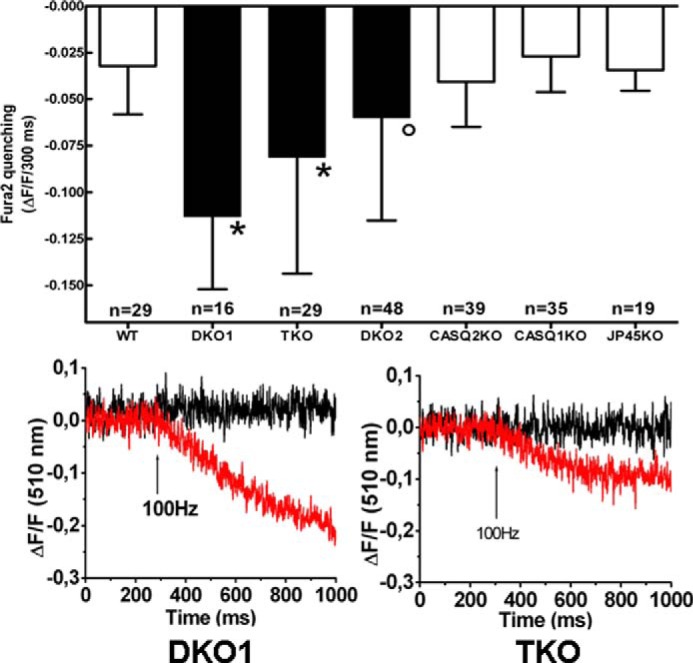
**Comparison of ECCE in FDB fibers.** *Upper panel*, fura-2 manganese quenching was carried out as described under “Experimental Procedures.” Values are the mean ± S.D. *n* indicates the number of FDB fibers investigated. *, multiple comparison Dunnett's ANOVA post test: DKO1 *versus* WT, *p* < 0.01; DKO1 *versus* CASQ2 KO, *p* < 0.01; DKO1 *versus* CASQ1 KO, *p* < 0.01; DKO1 *versus* JP45 KO, *p* < 0.01; TKO *versus* WT, *p* < 0.01; TKO *versus* CASQ2 KO, *p* < 0.01; TKO *versus* CASQ1 KO, *p* < 0.01; TKO *versus* JP45 KO, *p* < 0.01. °, multiple comparison Dunnett's ANOVA post test: DKO2 *versus* WT, *p* < 0.05; DKO2 *versus* CASQ1 KO. *p* < 0.05. *Lower panel*, fura-2 manganese quenching was carried out as described under “Experimental Procedures.” Single fibers were perfused with Tyrode's solution in which 1.8 mm CaCl_2_ was replaced with manganese and then stimulated by a 300-ms duration train of pulses delivered at 100 Hz. The *red* and *black traces* represent recordings carried out in the absence and in the presence of 50 μm nifedipine in the extracellular buffer, respectively. Representative traces of experiments of fibers isolated from four to six mice of each genotype are shown.

To evaluate the contribution of each individual genotype on the activation of excitation-coupled calcium entry, we compared Mn^2+^ quenching in FDB fibers from six different genotypes, namely WT, JP45 KO, CASQ1 KO, CASQ2 KO, JP45-CASQ1 double KO, JP45-CASQ2 double KO, and JP45-CASQ1-CASQ2 triple KO. The comparison of ECCE between different genotypes shows that the enhancement of ECCE requires the ablation of both CASQs and JP45 (Fig. 5). Indeed, we found that the values of Mn^2+^ quenching (Δ*F*/*F_o_*/300ms) in FBD fibers from DKO1 and TKO are significantly higher compared with those obtained in FDB fibers from WT, CASQ1 KO, CASQ2 KO, and JP45 KO mice (Fig. 5, *multiple comparison Dunnett's ANOVA post test *p* < 0.01). DKO2 display higher values compared with single KO mice; however, statistical significance was observed against the values of the fibers from WT and CASQ1 KO mice (Fig. 5, °, multiple comparison Dunnett's ANOVA post test; *p* < 0.05).

##### JP45 Interacts with Both CASQ1 and CASQ2 and the Interaction Is Calcium-dependent

**FIGURE 6.**
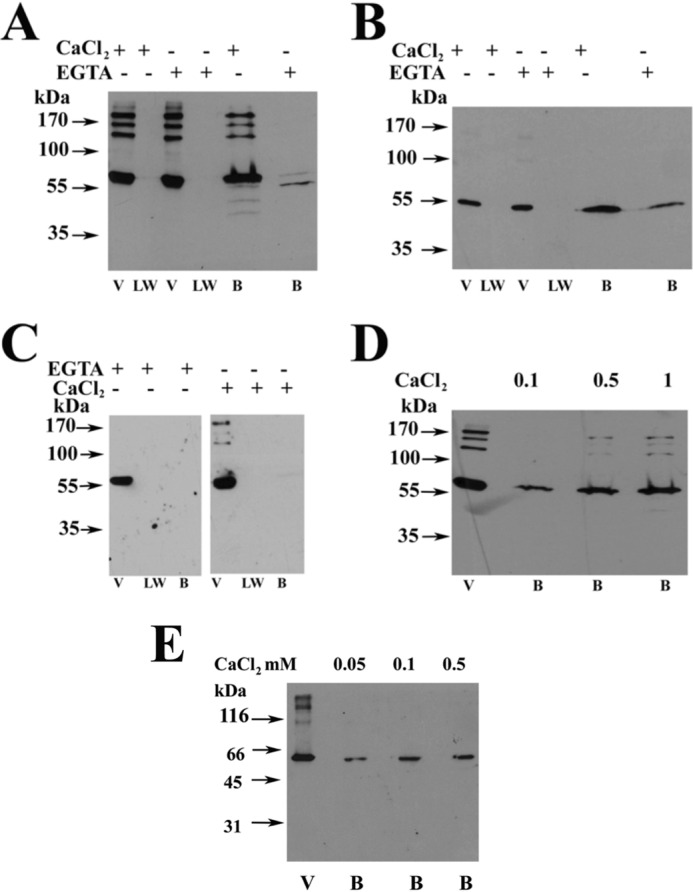
**Calcium dependence of JP45-CASQ interaction.** *Panels A*, *B*, *C*, and *D* show representative immunoblots of three to four experiments carried out with two different total SR preparations. *Panels A* and *B*, GST-JP45 fusion protein encompassing JP45 C-terminal domain (amino acid residues 149–332) was bound to glutathione-Sepharose beads and incubated with solubilized protein from skeletal muscle sarcoplasmic reticulum membrane fraction (*panel A*) and with solubilized protein from cardiac sarcoplasmic reticulum membrane fraction (*panel B*) as described under “Experimental Procedures” in a buffer containing 50 mm Tris-HCl, pH 8.5, 1 mm DTT, 20 mm NaCl, 0.1% CHAPS in the presence of 1 mm CaCl_2_ or 1 mm EGTA. Proteins present in the void (*V*), last wash (*LW*), and bound to the beads (*B*) were separated on a 10% SDS-PAGE, transferred onto nitrocellulose, and stained with anti CASQ1 Ab (*panel A*) or anti CASQ2 Ab (*panel B*). *Panel C*, pulldown experiments were performed by incubating GST protein with a skeletal muscle sarcoplasmic reticulum membrane fraction as described under “Experimental Procedures” in a buffer containing 50 mm Tris-HCl, pH 8.5, 1 mm DTT, 20 mm NaCl, 0.1% CHAPS in the presence of 1 mm CaCl_2_ or 1 mm EGTA. The Western blot was stained with anti CASQ1 Ab. *Panel D*, pulldown of solubilized protein from skeletal muscle SR membrane with GST-JP45 C-terminal domain fusion protein was carried out in the presence of 130 mm KCl, 20 mm NaCl plus increasing concentrations of Ca^2+^ (0.1, 0.5, and 1 mm). *V* = void; *B* = bound. The Western blot was stained with anti-CASQ1. *Panel E*, pulldown of solubilized protein from cardiac SR membrane with GST-JP45 C-terminal domain fusion protein was carried out in the presence of 130 mm KCl, 20 mm NaCl plus increasing concentrations of Ca^2+^ (0.05, 0.1, and 0.5 mm). *V* = void; *B* = bound. Proteins present in the void (*V*) and bound to the beads (*B*) were separated on a 10% SDS-PAGE, transferred onto nitrocellulose, and stained with anti-CASQ2 Ab.

**FIGURE 7.**
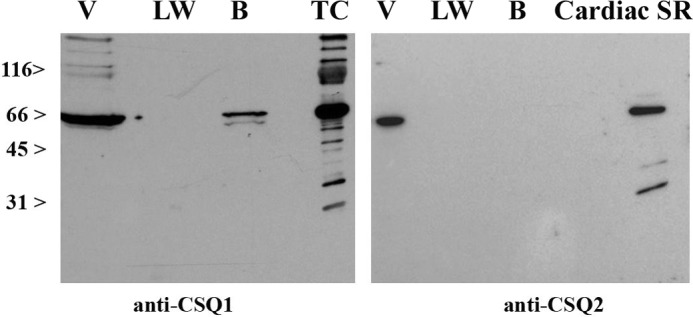
**Effect of high ionic strength on JP45-CASQs isoforms interaction.** Pulldown assays were carried out as described Fig 6, *panels A* and *B*. Solubilization of membrane fraction proteins and interaction with GST-JP45 fusion protein was carried out as described under “Experimental Procedures” in the presence of 1 m NaCl. *V*, void; *LW*, last wash; *B*, bound to the GST-JP45 fusion protein glutathione-Sepharose beads. Shown are representative immunoblots of three experiments carried out with two different total SR preparations. *Left panel*, staining of the blot membrane with anti-CASQ1 Ab. *Right panel*, staining of the blot membrane with anti-CASQ2 Ab. *TC*, skeletal muscle terminal cisternae fraction.

The activation of ECCE induced by the ablation of CASQ2 in a JP45 KO background suggests that JP45, in addition to CASQ1, is also able of interacting with CASQ2. Thus, in the next set of experiments we verified whether JP45 interacts with CASQ2. We carried out pulldown experiments by exposing a GST-tagged fusion protein of the C-terminal domain of JP45 to CHAPS-solubilized total SR fraction isolated from mouse heart and skeletal muscle. We found that at low ionic strength, the GST-C-ter-JP45 fusion protein interacts with both CASQ1 (Fig. 6, *panel A*) and CASQ2 (Fig. 6, *panel B*). The interaction of JP45 with both CASQ isoforms is optimal at 1 mm Ca^2+^, whereas at nanomolar calcium concentrations the binding activity of both CASQ isoforms to JP45 is close to zero. In the presence of 1 mm Ca^2+^, C-terminal domain of JP45 pulldown proteins had a molecular mass ranging from 130 to 170 kDa. These proteins stained by anti-CASQs Ab represent CASQ oligomers (31). We believe that the interaction of the GST-JP45 fusion protein with CASQs is specific as we did not observe any signal when we used the GST protein alone as bait in the pulldown assay (Fig. 6, *panel C*). We next examined the calcium dependence of JP45-CASQ1 interaction in the presence of 130 mm KCl and 20 mm NaCl, *i.e.* at cation concentrations similar to those of the intracellular milieu. Under these experimental settings, we found that maximal binding activity occurred at 1 mm Ca^2+^, whereas at 100 μm Ca^2+^, a Ca^2+^ concentration that is reached in the lumen of the sarcoplasmic reticulum of FDB fibers after depolarization-induced calcium release (32, 33), most of CASQ1 dissociates from JP45 (Fig. 6, *panel D*). JP45-CASQ2 interaction exhibits similar calcium dependence (Fig. 6, *panel E*). These results suggest that the binding of JP45 to CASQs most likely is not mediated by an electrostatic interaction via surface-exposed negatively charged amino acid residues of the calcium-free random coil form of CASQ monomers but rather requires calcium induced polymerization of CASQ molecules (34). In the next experiment we tested this hypothesis by performing CASQ pulldown assays under high salt conditions, a setting that promotes the monomerization of CASQs. We found that 1 m NaCl strongly reduced the interaction of JP45 with CASQ1 (Fig. 7, *left panel*) and abolished the interaction with CASQ2 (Fig. 7, *right panel*). Because CASQs form a calcium-dependent complex with JP45 in the presence of physiological concentrations of KCl and NaCl, one would expect that the depletion of JP45-CASQ2 JP45-CASQ1 complexes may facilitate excitation-coupled calcium entry in soleus muscles to support peak force development during repetitive tetanic stimulation. In the next set of experiments we examined this possibility by comparing the mechanical properties of soleus muscles from WT and TKO mice.

##### Tetanic Contraction of Soleus from WT and TKO Mice

**FIGURE 8.**
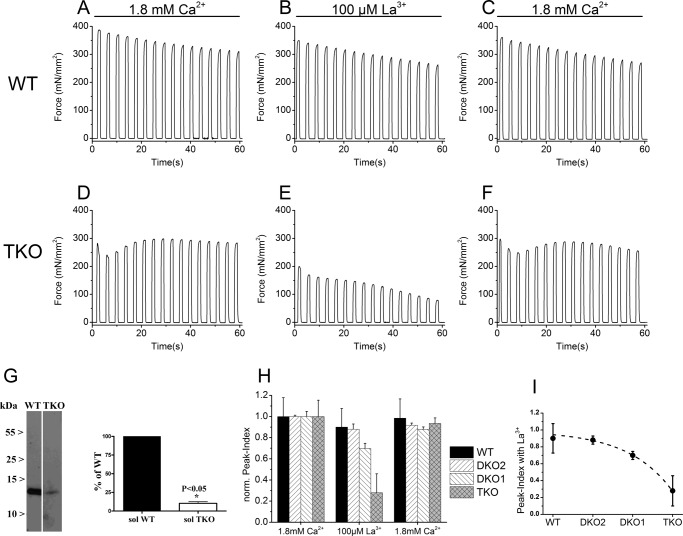
**Tetanic contraction of Soleus from WT/DKO1/DKO2 and TKO mice.** *Panels A–F*, traces of a train of tetanic contractures in wild-type and TKO carried out according the protocol described under “Experimental Procedures.” Shown is a representative response of 9 muscles from 10 mice. *Panel G*, Western blot of 37.5 μg of total homogenate of soleus from WT and TKO stained with anti-parvalbumin Ab. Histograms report values (mean ± S.D.) from four to six determinations from three different total homogenate preparations. Values are the mean ± S.D. *, *p* value of unpaired Student's *t* test. *Panel H*, normalized peak indexes of four strains. Values are the mean ± S.D. of 4–6 muscles from 2 or 3 mice; in the presence of 100 μm La^3+^ the overall ANOVA *p* values are <0.0001; multicomparison Dunnett's ANOVA post test: WT *versus* DKO2, *p* > 0.05; WT *versus* DKO1, *p* < 0.05; WT *versus* TKO, *p* < 0.05. *Panel I*, exponential curve fitting of the values of the peak indexes in the presence of La^3+^ values, which are reported in *panel H*.

The peak maximal tetanic contracture measured in the presence of 1.8 mm CaCl_2_ in the extracellular medium and normalized per muscle cross-sectional area of soleus from WT and TKO were not significantly different (327.86 ± 117.53 *versus* 364.64 ± 76.22 mN/mm^2^, respectively; mean ± S.D., *n* = 9) (where mN stands for millinewtons). The lack of difference of the onset specific tetanic force generated between the soleus from WT and TKO despite a lower calcium transient induced by field stimulation might be linked to differences in the myoplasmic calcium buffering capacity. We thus assessed the expression level of parvalbumin in total homogenates of soleus from WT and TKO mice. We found that there is ∼90% less parvalbumin in the soleus from the TKO (Fig. 8 *panel G*). According to the current models of calcium signaling in skeletal muscle fibers (35), the decrease of myoplasmic calcium buffering capacity, due to low expression of parvalbumin, is likely to be an adaptive event that may account for the lack of difference in the onset-specific tetanic force generated induced by field stimulation between WT and TKO. To the best our knowledge we think that it is unlikely that the decrease in parvalbumin influences ECCE in TKO muscles. Ablation of CASQ1 and CASQ2 in a JP45 KO background has a remarkable effect on the peak tension after repetitive tetanic contractions of 1100 ms duration delivered at 0.27 Hz. In the presence of 1.8 mm CaCl_2_ in the extracellular medium, we observed a slow linear decay of peak tetanic tension in solei from WT, which after 60-s peak tetanic tension, is 20% lower compared with that at the beginning of the repetitive tetanic contractures (Fig. 8, *panel A*). Solei from TKO mice show a rapid decrease of peak tetanic tension during the first couple tetanic stimulations. This brief phase is followed by a steady increase of peak tetanic tension, in that after 60 s peak tetanic tension is ∼6% lower compared with that observed at the beginning of the repetitive tetanic contractures (Fig. 8, *panel D*). The lower decrease of the peak tetanic tension in solei from TKO mice may be consistent with an enhanced calcium influx component that causes accumulation of intracellular calcium to support repetitive tetanic contractures. We tested this possibility by measuring repetitive tetanic contractures in the presence of 100 μm La^3+^ in the extracellular medium. La^3+^ caused a 10% decrease of peak tetanic tension at the end of the train of tetanic contractures in solei from WT mice (Fig. 8, *panel B*). The outcome is dramatically different in solei from TKO mice. In the latter case, 100 μm La^3+^ caused an 80% decrease of peak tetanic tension (Fig. 8, *panel E*). The effect of La^3+^ was reversed by washing the muscle with Tyrode's solution containing 1.8 mm CaCl_2_ (Fig. 8, *panels C* and *F*). We performed similar experiments with JP45-CASQ1 DKO1 and with JP45-CASQ2 DKO2. For simplicity of comparison between four different genotypes (WT, DKO1, DKO2, and TKO), we defined peak index as the ratio of the peak tetanic force after 1 min of repetitive tetanic contractures to that at the beginning; the peak index obtained in the presence of 100 μm La^3+^ in the extracellular medium was normalized to that obtained by incubating muscles in 1.8 mm CaCl_2_. The decrease of the normalized peak index in the presence of La^3+^ correlates with the extent of calcium influx into muscle fibers that is required to support tetanic tension during repetitive tetanic contractures and is strongly influenced by the mouse genotype. In the presence of La^3+^, soleus muscles from WT and DKO2 mice display similar peak index values, which indicates an average 10% decrease of the peak tetanic tension at the end of 60-s repetitive tetanic contractures (WT *versus* DKO2 multiple comparison Dunnett's ANOVA post test *p* > 0.05) (Fig. 8, *panel H*). The response of DKO1 is consistent with a compensatory role of CASQ2 as suggested in our previous experiments (Fig. 1), as the values of the normalized peak index are positioned at values (0.69 ± 0.04, mean ± S.D. *n* = 6; WT *versus* DKO1 multiple comparison Dunnett's ANOVA post hoc test *p* < 0.05) similar to those of WT and DKO2 mice rather than to those of TKO mice. Peak tetanic tension development of soleus from TKO mice strongly depends on calcium influx because 100 μm La^3+^ caused an exponential drop of the normalized peak index to 0.27 ± 0.17 (mean ± S.D. *n* = 6; WT *versus* TKO, multiple comparison Dunnett's ANOVA post hoc test *p* < 0.05) (Fig. 8, *panel I*). The effect of La^3+^ on the peak index in all genotypes could be reversed by washing out La^3+^ by incubating soleus muscles with Krebs containing 1.8 mm CaCl_2_ (Fig. 8, *panel H*).

## Discussion

In this study we document the role of the JP45-CASQ1 and JP45-CASQ2 complex on calcium entry in FDB fibers and soleus muscles. Our results on FDB from TKO mice show that calcium transients induced by tetanic stimulation rely on massive calcium influx via La^3+^- and nifedipine-sensitive calcium channels. The massive calcium influx is crucial to maintain muscle force development in soleus muscles and suggests that both JP45-CASQ1 and JP45-CASQ2 complexes are important modulators of this calcium entry in slow fibers. In addition, we demonstrate that the interaction between CASQ isoforms and the C-terminal domain of JP45 occurs at calcium concentrations similar to those present in the lumen of the sarcoplasmic reticulum in resting condition and that JP45 dissociates from both CASQs isoforms at free Ca^2+^ concentrations similar to those present in the SR lumen after depolarization-induced calcium release.

### 

#### 

##### Role of JP45-CASQ1 and JP45-CASQ2 Complexes in Tetanic Tension Development of Slow Twitch Muscles

The development and maintenance of peak tetanic tension in soleus muscle despite the triple ablation of junctional sarcoplasmic reticulum proteins is surprising and may result from the activation of more than one adaptive mechanism. The data of the present study provide insights as to one such possible mechanism(s). Our study demonstrates that the ability of soleus muscles to develop and maintain isometric force during and at the end of repetitive tetanic stimulation in the absence of JP45-CASQ1 and JP45-CASQ2 complexes is due to the activation of excitation-coupled calcium entry via La^3+^-sensitive calcium channels and/or via nifedipine-sensitive calcium channels as shown by Mn^2+^ quenching experiments. This adaptive mechanism is dynamic as its extent is different among the different mouse models. Indeed, the comparison of the normalized peak indexes shows that the activation of the La^3+^-sensitive calcium entry is small after the ablation of JP45-CASQ2 complex; however, its contribution is more relevant in the JP45-CASQ1 null mice. The decrease of the normalized peak index in soleus from JP45-CASQ1 mice is lower than expected if one takes into account that 100% of soleus fibers express the CASQ1 isoform. The relative mild decrease of the peak index in soleus from DKO1 mice could be due to the compensatory expression of the JP45-CASQ2 complex in 40% of the type I fiber of soleus. The decrease of the peak index in the presence of La^3+^ obtained with soleus from TKO mice is not linear and may result from unique adaptive mechanisms that are determined by the chronic ablation of three junctional sarcoplasmic reticulum proteins. However, we cannot exclude the possibility of a synergy between JP45-CASQ1 and JP45-CASQ2 complexes in the modulation of calcium entry channels in slow muscles. The non-linearity of the decrease of the peak index of soleus from TKO mice may in fact be consistent with the removal of an allosteric inhibition of the JP45-CASQs complexes on calcium entry channels. In particular, our data show that the ablation of JP45-CASQ2 complex has *per se* a small inhibitory effect on calcium entry. However, the removal of the JP45-CASQ2 complex on a JP45-CASQ1 background results in a positive cooperative effect on activation of nifedipine- and La^3+^-sensitive calcium entry channels in soleus from TKO mice during the repetitive tetanic contractures in presence of 1.8 mm Ca^2+^ in the extracellular medium (Fig. 8, *panel I*).

##### JP45-CASQs Complex Is a Negative Regulator of the Excitation-coupled Calcium Entry

Skeletal muscle fibers express two calcium entry mechanisms: 1) calcium entry via Ca_v_1.1, also referred to as excitation coupled calcium entry (ECCE), which is triggered either by a train of action potential or by prolonged depolarization (6, 7); 2) calcium entry activated by depletion of sarcoplasmic reticulum store (SOCE, store operated calcium entry) mediated by Stim and Orai1 (36–38). ECCE requires the expression of both Ca_v_1.1 and RyR1 on the T Tubule membrane and junctional sarcoplasmic reticulum, respectively, and the protein-protein interaction between RyR1 and Ca_v_1.1 is thought to be of paramount importance for ECCE activation (7). The conformation state of both RyR1 and Ca_v_1.1 is also modulated by the interaction with a number of accessory proteins, including JP45 and CASQs, which might in turn affect ECCE. In this study we dissected the role of the JP45 and CASQ on the molecular machinery involved in the modulation of ECCE by comparing ECCE in FDB fibers isolated from six different knock-out mouse models, namely WT, CASQ1 KO, CASQ2 KO, JP45 KO, JP45-CASQ1 double KO, JP45-CASQ2 double KO, and JP45-CASQ1-CASQ2 triple KO. Our results clearly show that ablation of each individual gene has little effect on ECCE activated by a train of action potentials delivered at 100 Hz for 300 ms. On the other hand, the activation of ECCE appears to be enhanced by the ablation of JP45-CASQ1, JP45-CASQ2 complexes, or by the ablation of both as in the case of TKO mice. These results imply that JP45-CASQs complexes act as negative regulators of ECCE. The extent of ECCE activation in FDB fibers from DKO2 is lower compared with that of DKO1. These results most likely reflect (i) the low percentage of type 1 fiber expressing CASQ2 in our FDB preparation and (ii) the co-expression of the JP45-CASQ1 complex in the type 1 fibers, which may in turn maintain an inhibitory signal on ECCE activity. Further experiments are needed to establish the exact molecular mechanism by which the JP45-CASQs complex acts as a negative regulator of ECCE.

##### Calcium Dependence of JP45-CASQs Interactions

In the present report we also show that the interaction between JP45 and CASQs isoforms is calcium-dependent; namely maximal JP45 calsequestrin interaction occurs at Ca^2+^, K^+^, and Na^+^ concentrations similar to those present in the sarcoplasmic reticulum lumen under resting conditions. The junctional face membrane is also endowed with two other well characterized calsequestrin interacting proteins, namely junctin and triadin. The calcium dependence of interaction of JP45 with calsequestrin appears to be the opposite compared with that of junctin and triadin. The latter proteins exhibit optimal calsequestrin interaction activity in the presence of nanomolar calcium (39, 40). In a previous study we showed that JP45 is enriched in the sarcoplasmic reticulum junctional face membrane because its subcellular membrane distribution is identical to that of RyR and calsequestrin (41). Although we did not measure the relative affinity of calsequestrin for junctin/triadin *versus* that of JP45, the present study suggests that in the resting state the major protein anchoring calsequestrin to the skeletal sarcoplasmic reticulum junctional face membrane is JP45. In resting conditions most likely the polymer form of calsequestrin interacts with JP45, and the depolymerization of calsequestrin caused by the decrease of calcium concentration in the lumen of the terminal cisterna during calcium release may cause its dissociation from JP45. Although the exact functional relevance of such a potential dynamic interaction between JP45 and calsequestrin on the EC-coupling mechanism remains elusive, we cannot exclude the possibility that the dissociation of JP45 from CASQs may affect its interaction with DHPR.

## Author Contributions

M. D., R. B., and S. G. P. provided CASQ2 KO mice. F. P. provided CASQ1 KO mice. B. M., J. E., S. T., L. B., and F. Z. carried out the experiments. S. T. and F. Z. designed the experiments and wrote the manuscript.

## Supplementary Material

Supplemental Data

## Footnotes

3 B. Mosca, J. Eckhardt, L. Bergamelli, S. Treves, R. Bongianino, M. De Negri, S. G. Priori, F. Protasi, and F. Zorzato, unpublished observations.

## Footnotes

2 The abbreviations used are:

SR: sarcoplasmic reticulum
SERCA: sarco(endo)plasmic reticulum calcium ATPase
ECCE: excitation-coupled calcium entry
RyR: ryanodine receptor
CASQ: calsequestrin
FDB: flexor digitorum brevis
EDL: extensor digitorum longus
DKO: double KO
TKO: triple KO
Ab: antibody
ANOVA: analysis of variance.
