# Supplementary material for: Role of the JP45-Calsequestrin Complex on Calcium Entry in Slow Twitch Skeletal Muscles
Source: J Biol Chem. 2016 May 4;291(28):14555–65. doi: 10.1074/jbc.M115.709071 (PMC4938177; doi:10.1074/jbc.M115.709071)
Supplement: Supplemental Data [file supp_291_28_14555__index.html]

Role of the JP45-calsequestrin complex on calcium entry in slow twitch skeletal muscles — Role of the JP45-calsequestrin complex on calcium entry in slow twitch skeletal muscles — Role of the JP45-Calsequestrin Complex on Calcium Entry in Slow Twitch Skeletal Muscles — JP45 and Calsequestrin Complex — Supplemental Data 

# Role of the JP45-Calsequestrin Complex on Calcium Entry in Slow Twitch Skeletal Muscles

## Supplemental Data

- Supplementary Figure 1 (.pdf, 255 KB) - Supplementary Figure1
