# Supplementary material for: Role of the JP45-Calsequestrin Complex on Calcium Entry in Slow Twitch Skeletal Muscles
Source: J Biol Chem. 2016 May 4;291(28):14555–65. doi: 10.1074/jbc.M115.709071 (PMC4938177; doi:10.1074/jbc.M115.709071)

Supplementary Figure 1

Representative western blots on total SR preparations from WT mice stained with the specified antibodies: anti-RyR1, 7.5 µg SR protein; anti-Cav1.1, 10 µg SR protein ; anti-JP45, 10 µg SR protein ; anti-SERCA1, 5 µg SR protein; anti-SERCA2, 5 µg SR protein ; anti-calreticulin, 20 µg SR protein; anti-sarcalumenin, 10 µg SR protein ; anti-albumin, 5 µg SR protein ; anti-beta1, 10 µg SR protein. Immunostaining was performed as described in the methods section.

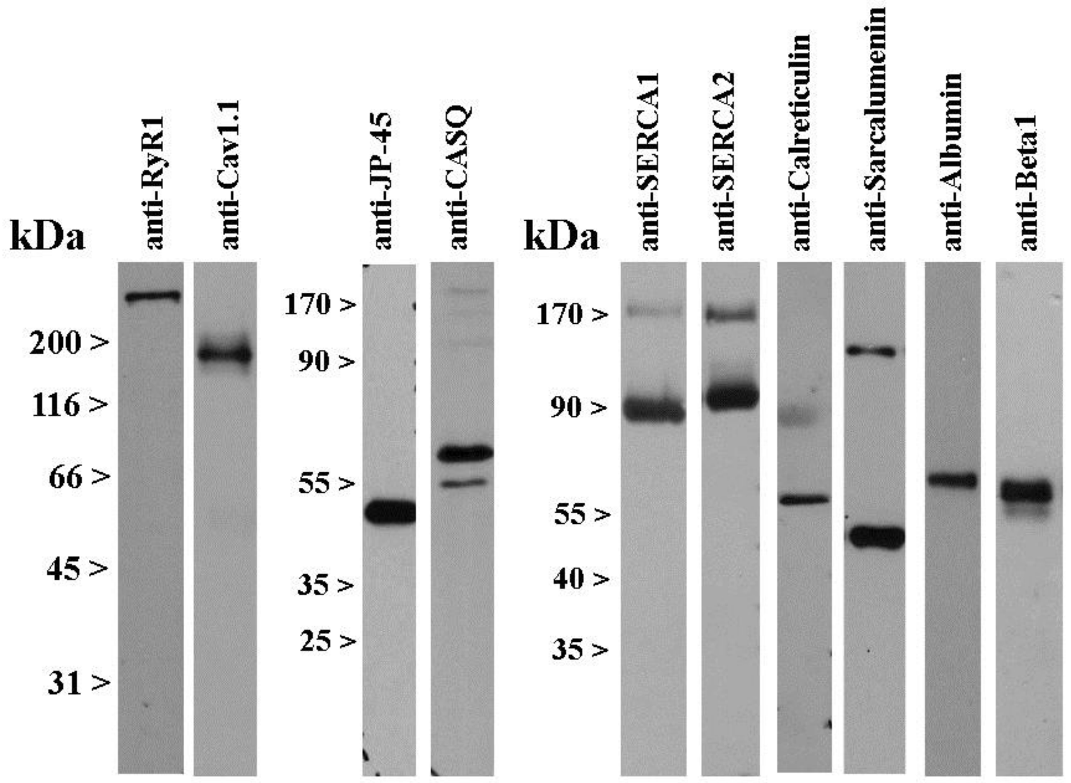

Supplement: Supplemental Data [file 10.1074_M115.709071_jbc.M115.709071-1.pdf]
